# Exploration of Seafarers’ Mobile Proficiency as a Prerequisite for Possible Health App-based Health Promotion on Board

**DOI:** 10.1177/00469580231206264

**Published:** 2023-11-01

**Authors:** Luciano Cemil Arslan, Dorothee Dengler, Lukas Belz, Felix Alexander Neumann, Birgit-Christiane Zyriax, Volker Harth, Marcus Oldenburg

**Affiliations:** 1 Maritime Medicine, Institute for Occupational and Maritime Medicine Hamburg (ZfAM), University Medical Center Hamburg-Eppendorf (UKE), Hamburg, Germany; 2 Preventive Medicine and Nutrition, Midwifery Science - Health Services Research and Prevention, Institute for Health Services Research in Dermatology and Nursing (IVDP), University Medical Center Hamburg-Eppendorf (UKE), Hambur

**Keywords:** mobile proficiency, seafarers, health apps, maritime, health intervention, health promotion

## Abstract

Numerous health hazards characterize the profession of a seafarer. The job-specific environment may affect the crews’ well-being and mental health. The options for health promotion are limited, as seafarers are isolated for long periods and are a difficult-to-reach collective. A digital app-based health management system might offer a promising approach in this regard. This study aims to identify seafarers’ technical competencies as a prerequisite for possible health app-based health promotion. A total of 976 seafarers (response rate 75.1%) on 65 ships of a Hamburg shipping company completed the standardized questionnaire MDPQ-16 (Mobile Device Proficiency Questionnaire) that assesses the IT competence in 8 different sub-scales. The results were stratified in respect to occupational groups aboard as well as compared to a reference collective ashore. The seafarers had an MDPQ-16 sum-score of 4.40 (SD 0.84), scoring 0.21 points higher than the land-based reference collective. Significant differences were observed between the ratings and officers in almost every sub-scale and item of the MDPQ-16. The highest sum-score was held by the nautical officers with 4.71. Greater differences between officers and ratings were observed in sub-scales related to rather complex tasks. The seafarers demonstrated a high level of IT proficiency, even surpassing the land-based sample, suggesting that their technical competence is adequate for app-based health promotion. In view of the higher scores of officers, the selection of health apps has to be tailored to the seafarers’ different levels of mobile competence in order to maximize the improvement in mental health and well-being. Further research is needed to determine seafarers’ needs and interests.

**What do we already know about this topic?**
There is a limited amount of existing research on digital health management specifically focusing on seafarers. Consequently, there is little knowledge on this topic at present. However, it is worth noting that health apps present a promising approach to address the challenges of health promotion in the demanding maritime environment.
**How does your research contribute to the field?**
The present research fills a significant gap in the literature by examining whether health promotion among seafarers via health apps might encounter any specific barriers or challenges due to the necessary knowledge and requirements. Through this study, it is aimed to assess the feasibility and potential of implementing health promotion interventions using health apps within this population. The findings of this research shed light on the competence of seafarers in utilizing mobile technology and highlight the potential for app-based health interventions.
**What are your research’s implications toward theory, practice, or policy?**
The study provides insights into the digital health competence of seafarers and emphasizes the viability of implementing health interventions through the use of mobile health apps. These findings can inform the development of tailored health app programs to promote and enhance the health and well-being of seafarers. Further research is needed to explore the current usage patterns, as well as the level of interest and acceptance among seafarers, in order to optimize the success of health promotion initiatives.

## Introduction

Seafaring is a profession that poses a multitude of physical and psychosocial health risks, some of which are linked to occupation-based hazards, while others are associated with lifestyle behavior. Common psychosocial stressors include the exposition to permanent physical influences (such as noise and swell) as well as isolation, separation from family, loneliness on board, shift work, and reduced sleep quality and duration.^1-3^ Consequently, seafarers frequently experience sleep deprivation, fatigue, and psychophysical exhaustion.^4-6^

The literature indicates that the profession also seems to be associated with an increased risk of developing cardiovascular diseases (CVD) and having CVD-associated risk factors such as high blood pressure, obesity, and elevated blood lipids.^7-12^ Seafarers also seem to be a population with an elevated risk for smoking as a maritime study found out that Danish seafarers smoke distinctly more cigarettes compared to the general Danish male population.^13^

Furthermore, seafarers’ access to a well-balanced diet at sea is often limited due to job-specific conditions such as logistics, transport, and costs that pose challenges to maintaining healthy nutrition on board.^14^ Specifically, a survey conducted among European and Asian seafarers reported that less vegetable, fruit and fish, but more coke and sausage were consumed at sea compared in respect to the diet at home.^15^ Reduced physical activity on board is another health-impairing factor among seafarers described in the literature. For instance, seafarers exhibit reduced physical activity while navigating on high seas compared to shore leave at home. A majority of 70% of the participants engage in physical exercise at least 2 times per week while on land, in comparison to only 39% while onboard.^16^ According to the authors fewer than 20% of crew members use fitness equipment when it is accessible on board.

Consequently, the demanding work environment and personal living conditions on board make health promotion a vital issue for seafarers. The authors of a Norwegian study argue that there has been limited research on health promotion and a lack of evidence supporting the effectiveness of health promotion measures in maritime settings.^17^ Typically, previous maritime health interventions have focused on certain target groups or health risks, neglecting health promotion topics that would lead to broader changes in behavioral patterns and some issues entirely.^17,18^ This view is sustained by a systematic review that examined 10 intervention studies aimed at lifestyle changes and health outcomes through educational and structural interventions in a maritime setting.^19^ The review revealed that most of the intervention studies were poorly designed and implemented, with limited methodological quality. According to 2 recent studies,^17,19^ this is due to the maritime environment that poses unique challenges for implementing successful and sustainable health promotion on board. In light of the described shipboard challenges, the maritime setting is in high need for health promotion concepts for a remote working collective. A digital, health app-based intervention could provide an opportunity for specific, self-responsible health promotion in this area.

A systematic review of 20 scientific land-based surveys addressing the issue of health app effectiveness showed that 16 of the included studies demonstrated a positive impact on users’ health behavior or health.^20^ Moreover, a general interest in app-based health promotion might exist among seafarers as the findings of a recent maritime study suggest.^21^

App-based health intervention could offer individual solutions and pose a promising starting point for long-lasting behavioral changes as they can address lifestyle-associated factors such as unhealthy nutrition, physical inactivity or tobacco use, as well as risks arising from the occupational environment, such as stress. Additionally, measures could be carried out continuously on board and would not depend on the presence of health professionals on the ships or other external factors.

Basically, mobile proficiency is the basic requirement for app-based health promotion. There are no national or international empirical values or studies on how experienced multicultural crews are in dealing with health apps. Before starting a complex electronic health intervention on board, the question of IT proficiency of the crews needs to be clarified. This is the only way to avoid possible frustrating experiences amongst seafarers on the one hand and unnecessary costs for the shipping companies on the other. The approach allows to identify deficits in the use of apps at a good point in time. Furthermore, based on the results of this study, concrete IT-training courses for the ship’s crews can be designed. Provided a sufficient security in dealing with health apps the way will then be paved for an entertaining, instructive and thus successful health intervention via apps.

## Materials and Methods

### Data Collection

In June and July 2021, an electronic self-administered questionnaire was employed to collect data from the ship crews of 65 merchant vessels of a German shipping company. The questionnaire encompassed demographic data, technical requirements for app-based health management, and the assessment of mobile proficiency. The demographic and lifestyle information were gathered using a set of 9 closed-ended questions about the *individual’s rank, age, seafaring experience, gender, family status, child status, smoking habits, daily cigarette consumption*, and *alcohol consumption*. Additionally, an open-ended question inquired about the respondent’s *nationality*. The amount of people that have already downloaded a health app (downloader) was assessed with 1 closed question.

The mobile proficiency of seafarers was assessed using the Mobile Device Proficiency Questionnaire (MDPQ-16),^22^ which was developed to assess individuals’ ability to perform various operations on a smartphone or tablet. The MDPQ-16 is based on 16 different activities on a mobile device (eg, *“I can navigate onscreen menus using the touchscreen”*), which are divided into 8 sub-scales (*Mobile device basics; Communication; Data and file storage; Internet; Calendar; Entertainment; Privacy; Software management*). The questions within a sub-scale are combined into a sum-score using principal components analysis (*PCA*) so that a single factor is representatively identified for each sub-scale. The closed response options consist of a 5-point scale with which the respondents could assess their IT skills (eg, *1 = never tried, 2 = not at all, 3 = not very easily, 4 = somewhat easily, 5 = very easily*), with a low score meaning a low proficiency. The reliability of the MDPQ-16 was found to be very high (*Cronbach’s α = .96*). The reliability of the individual sub-scales ranged from 0.75 to 0.99 (*mobile device basics, α = .94; communication, α = .83; data and file storage, α = .89; internet, α = .99; calendar, α = .98; entertainment, α = .75; privacy, α = .87; software management, α = .89*).^22^ Two further studies identified Cronbach’s α of .99 for the MDPQ-16 as well.^23,24^ Consistent with these results, a high Cronbach’s α of .96 was calculated for the present study.

The results of the MDPQ-16 were compared to the study sample of Moret-Tatay et al.^23^ The study was selected as a control group for several reasons. A thorough literature review revealed it to be the cohort with the highest number of participants. In addition, it had investigated the largest proportion of subjects under the age of 65, thereby rendering it the most suitable cohort for comparison with the one investigated in this study. Furthermore, it represents the most recent utilization of the MDPQ-16, making it a contemporary benchmark and better reflecting the rapidly evolving digital scenery.

Since our study sample consisted only of seafarers younger than 65 years old, the seafaring sample was compared to the available land-based group of Moret-Tatay et al^23^ below 65 years. Their land-based sample consisted of 407 participants with 132 participants aged 19 to 34, 116 participants aged 35 to 64, and 159 participants with 65+ years (who were excluded in the present comparison). The mean age was 53.1 (SD 23.2). The sample consisted of 60.5% women.

The completion of the questionnaire was entirely voluntary. At the commencement of the survey, the participants were notified through an introductory text that the questionnaire was anonymous and optional. Their informed consent was obtained. The seafarers were granted 21 days to complete the questionnaire. The ethics committee of the Hamburg Medical Association approved the study and gave a positive ethics vote (*PV7174-4572-BO-ff*).

### Study Population

The questionnaire was handed out to 1300 seafarers on 65 merchant vessels. No exclusion criteria were applied. A sample size estimation revealed a minimum participation of 279 persons. Nine hundred seventy-six seafarers answered the questionnaire (response rate 75.1%). Of these, 2 seafarers (0.2%) were 19 years old or younger, 196 were between the ages of 20 and 29 (21.6%), 302 were between the ages of 30 and 39 (33.3%), 263 were between the ages of 40 and 49 (30.0%), 119 were between the ages of 50 and 59 (13.1%), and 26 reported being 60 years old or older (2.9%). Six (0.6%) crew members were female.

The study population was multicultural, with 207 (27.2%) Europeans and 553 (72.8%) non-Europeans. Among the surveyed seafarers, Filipinos were the most represented, with 502 (66.1%), followed by 60 Ukrainians (7.9%) and 54 Romanians (7.1%). 638 (68.8%) of the seafarers reported having children. Two hundred ten of the study population were single (22.8%), 696 were married (75.5%), and 16 divorced (1.7%). A total of 584 seafarers (66.7%) were non-smokers, and 69 (7.9%) reported that they had quit smoking. On average, the seafarers smoked 12.2 cigarettes per day (SD 7.0). Among the respondents, 355 held an officer’s rank (38.9%) and 557 (61.1%) were ratings. The rank-related demographic data of the seafarers who had answered the question targeting this information are presented in Table 1, displaying the total sample of seafarers (912; excluding 64 seafarers, who did not answer this question) and the 2 subsamples of officers and ratings. Furthermore, the questionnaire revealed that the participating seafarers had 13.8 (SD 9.1) years of seafaring experience and had been on their respective vessels for 5.3 (SD 3.12) months.

**Table 1.** Demographic Data Distinguished by Occupation and Ranks.

Characteristics	Officers (n = 355)	Ratings (n = 557)	Total sample (N = 912)^a^
Ranks, n	201 nautical officers, 154 technical officers	284 ratings on deck, 153 engine ratings, 79 galley staff, 31 cadettes, 10 others	201 nautical officers, 154 technical officers, 284 ratings on deck, 153 engine ratings, 79 galley staff, 31 cadettes, 10 others
Age, n (%)^b^			
<40 years	210 (60.3)	278 (51.7)	488 (55.1)
≥40 years	138 (39.7)	260 (48.3)	398 (44.9)
Gender, n (%)^b^			
Male	352 (99.4)	554 (99.5)	906 (99.5)
Female	2 (0.6)	3 (0.5)	5 (0.518)
Origin, n (%)^b^			
Europeans	187 (61.9)	19 (4.2)	206 (27.4)
Non-Europeans	115 (38.1)	432 (95.8)	547 (72.6)
Married, n (%)^b^	266 (77.1)	402 (73.9)	668 (77.4)
Children, n (%)^b^	227 (65.0)	386 (70.8)	613 (568.6)
Smoking status, n (%)^b^			
Never smoked	208 (62.1)	361 (70.4)	569 (67.1)
Former smoker	94 (28.1)	119 (23.2)	213 (25.1)
Smoker	33 (9.8)	33 (6.4)	66 (7.8)
Alcohol consumption, n (%)^b^			
More than 0.5 L beer, 0.3 L wine or 0.08 L liqueur per day	11 (3.6)	24 (5.7)	35 (4.8)

a 64 seafarers did not answer the question to the rank and are not displayed here.

b Differences in the categories’ absolute sum are due to respective missing data.

### Statistics

Statistical analysis was performed using the open-source program R (version 4.3.0) offered by the R Foundation for Statistical Computing, founded by the R Core Team. The percentages displayed relate to the number of respondents to the respective question and not to the entire sample. The statistical significance between data sets was tested with/by a Pearson’s Chi-Squared test or a Fisher’s exact test. Independency between individuals was assumed. Statistical testing was performed with an α risk equal to .05.

Crude odds ratio (OR) including 95% confidence intervals was calculated by binary logistic regression. Multivariate analysis was performed by linear logistic regression and was adjusted by the main demographic/occupational variables (age, rank, ethnicity).

Excluding the 6 female seafarers resulted in the same significant results, which is why they were kept in the data set.

## Results

### Mobile Proficiency in the Total Seafaring Collective

The examined sample had an MDPQ-16 sum-score of 4.40 (SD 0.84). The seafarers achieved the highest results in the “*Basics*” sub-scale (4.56; SD (0.86)) and “*Entertainment*” (4.53; SD (0.86)). The item with the highest score was “*I can listen to music”* (*Entertainment*; 4.65; SD (0.82)) and “*I can navigate onscreen menus using the touchscreen* “(*Basics*; 4.51; SD (1.01)). The seafarers had the lowest scores in the sub-scale “*Calendar*” (4.16; SD (1.21)) and “*Data/file storage*” (4.32; SD (1.15)). The items with the lowest scores were both items of the sub-scale “*Calendar*”: “*I can enter events and appointments into a calendar*” (4.13; SD (1.27)) and “*I can check the date and time of upcoming and prior appointments* “(4.19; SD (1.23)) (Table 2).

**Table 2.** MDPQ-16 Sum-Score and Sub-Scales Compared to the Reference Sample (<65 years).

	Seafarers (n = 976)		Reference sample^a^ <65 years (n = 248)		
	n	M (SD)	n	M (SD)	*P* ^*^
MDPQ-16 sum-score	918	4.40 (0.84)	248	4.19 (1.00)	.002
Basics	956	4.56 (0.86)	248	4.70 (0.44)	.001
Communication	960	4.37 (1.09)	248	4.39 (1.32)	.849
Data/file storage	965	4.32 (1.15)	248	3.94 (1.84)	.002
Internet	947	4.41 (0.94)	248	4.36 (1.23)	.533
Calendar	951	4.16 (1.21)	248	3.93 (2.15)	.093
Entertainment	950	4.53 (0.86)	248	4.11 (1.67)	<.001
Privacy	944	4.40 (0.98)	248	3.90 (1.70)	<.001
Software management	946	4.36 (1.08)	248	4.21 (1.51)	.124

a Sample by Moret-Tatay et al.^23^

* Fisher’s exact test, *P* < .05.

### Mobile Proficiency by Occupation and Ranks

Significant differences were found between officers and ratings within almost all sub-scales and items of the MDPQ-16, whereby the officers consistently scored higher (Table 3). The biggest differences were found in the sub-scales “*Communication*” (Δ 0.43) and “*Data/file storage*” (Δ 0.41). The items with the biggest differences were “*I can send pictures by email*” (*Communication*; Δ 0.47) and “*I can transfer information on my mobile device to my computer*” (*Data/file storage*; Δ 0.41). On the other hand, the 2 sub-scales and items with the smallest differences between officers and rankings were “*Entertainment*” (Δ 0.14) and “*Basics*” (Δ 0.22), and “*I can use the onscreen keyboard to type*” (*Basics*; Δ 0.14) and “*I can listen to music*” (*Entertainment*; Δ 0.04). The item “*I can listen to music*” was also the only item where no significant difference could be found.

**Table 3.** MDPQ-16 Distinguished by Occupation (Sum-Score, Sub-Scales and Items).

	Officers (n = 355)		Ratings (n = 557)		
I can. . .	n	M (SD)	n	M (SD)	*P* ^*^
MDPQ-16 sum-score	341	4.60 (0.69)	529	4.30 (0.88)	<.001
Basics	352	4.72 (0.70)	550	4.50 (0.89)	<.001
. . .navigate onscreen menus using the touchscreen	355	4.72 (0.81)	555	4.41(1.06)	<.001
. . .use the onscreen keyboard to type	352	4.71 (0.73)	551	4.57 (0.88)	.010
Communication	354	4.66 (0.78)	551	4.23 (1.16)	<.001
. . . send emails	355	4.70 (0.75)	555	4.31 (1.15)	<.001
. . .send pictures by email	354	4.62 (0.90)	552	4.15 (1.29)	<.001
Data/file storage	355	4.59 (0.88)	555	4.18 (1.24)	<.001
. . .transfer information on my mobile device to my computer	355	4.61 (0.86)	555	4.20 (1.26)	<.001
. . .transfer information on my computer to my mobile device	355	4.56 (0.94)	555	4.17 (1.26)	<.001
Internet	349	4.63 (0.74)	548	4.29 (0.99)	<.001
. . .find information about my hobbies and interests on the Internet	352	4.69 (0.73)	553	4.30 (1.04)	<.001
. . .find health information on the Internet	349	4.58 (0.86)	550	4.28 (1.07)	<.001
Calendar	351	4.39 (1.06)	551	4.04 (1.26)	<.001
. . .enter events and appointments into a calendar	352	4.37 (1.09)	553	3.99 (1.34)	<.001
. . .check the date and time of upcoming and prior appointments	351	4.40 (1.07)	552	4.08 (1.27)	<.001
Entertainment	350	4.62 (0.81)	550	4.48 (0.86)	.016
. . .use the device’s online “store” to find games and other forms of entertainment	353	4.56 (0.95)	551	4.31 (1.09)	<.001
. . .listen to music	350	4.68 (0.79)	553	4.64 (0.81)	.455
Privacy	348	4.56 (0.81)	546	4.31 (1.04)	<.001
. . .setup a password to lock/unlock the device	353	4.65 (0.80)	553	4.42 (1.06)	<.001
. . .erase all Internet browsing history and temporary files	348	4.47 (1.01)	547	4.19 (1.21)	<.001
Software management	346	4.55 (0.96)	549	4.28 (1.08)	<.001
. . .update games and other applications	348	4.52 (1.00)	549	4.24 (1.16)	<.001
. . .delete games and other applications	347	4.57 (0.97)	551	4.33 (1.12)	.001

* Fisher’s exact test, *P* < .05.

In terms of ranks, nautical officers had the highest MDPQ-16 sum-score with 4.71 (0.56), followed by cadets (4.68; SD (0.49)), galley staff (4.45; SD (0.76)), technical officers (4.44; SD (0.80)), engine ratings (4.26; SD (0.84)) and deck ratings (4.24; SD (0.95)). Between the nautical ranks (*nautical officers & deck ratings*) and the technical ranks (*technical officers & engine ratings*), no significant difference could be identified in regard to the sum-score and the sub-scales.

In the multivariate analysis, it was found that holding an officer’s rank (as opposed to a rating position) was more strongly associated with being of European nationality (adjusted odds ratio (aOR) 38.0; 95% confidence interval (CI) 23.10-65.70) and having previously downloaded a health app (aOR 1.68; 95% CI 1.48-2.59), and less strongly associated with being 40 years of age or older (aOR 0.63; 95% CI 0.42-0.93). No significant associations between ratings and officers were observed regarding the family status (single/divorced vs married).

### Mobile Proficiency by Demographic Data

Between Europeans and Non-Europeans, Europeans scored significantly higher in all sub-scales and all items (except for the item *“I can listen to music” (Entertainment*; Δ 0.08), where no significance and the lowest difference was found (*P* = .186)). The largest differences were found in the sub-scale “*Calendar*” (Δ 0.52; *P* < .001) and the item “*I can enter events and appointments into a calendar*” (*Calendar*; Δ 0.53; *P* < .001).

### Download Behavior

Four hundred seventy-eight seafarers stated already having downloaded at least 1 health app in the past (downloader) (52.5%). The mobile proficiency of seafarers who had already downloaded a health app (downloaders) was compared to crew members that haven’t (non-downloaders). In almost all items and sub-scales, significant differences in MDPQ-16 were found between downloaders and non-downloaders. Downloaders had a significantly higher score in each item, sub-scale, and sum-score, with the exception of the item “*I can use the onscreen keyboard to type*” (Δ 0.106; *P* = .062).

### Comparison With a Reference Group

Comparing seafarers with the reference group of Moret-Tatay et al23 the MDPQ-16 sum-score has a significant difference mas shown in Table 2, with the seafarers scoring higher with a difference of 0.21 points (*P* = .002). The seafarers also performed significantly better in the sub-scales “*Data/file storage*” (Δ 0.38), “*Entertainment*” (Δ 0.42), and “*Privacy*” (Δ 0.50). The only sub-scales in which the reference sample performed significantly better was the “*Basics*” sub-scale (Δ 0.14). No significant differences were found in any of the other sub-scales.

## Discussion

The present study aimed to evaluate insights on mobile proficiency among seafarers using a large sample size, resulting in highly reliable estimates of survey responses.

### Seafarers’ Data

This study examined the mobile device proficiency of seafarers as a required prerequisite for possible future app-based health intervention. The seafarers’ mobile proficiency sum-score, assessed with MDPQ-16, was 4.40 (SD (0.84)) with 5 being the highest possible score. As described in the literature,^22,23^ in this study sample a greater variation (SD) and lower scores could be identified on sub-scales related to more complex actions such as calendaring, and data and file storage, whereas higher scores were more prominent among the simpler and probably more frequently performed actions such as “*Basics*” or “*Entertainment*.” The highest score was found in the item “*I can listen to music*” (*Entertainment*; 4.65; SD (0.82)). This is also where the lowest variation (SD) was found.

As shown also by Roque and Boot^22^ and Moret-Tatay et al,^23^ older seafarers demonstrated lower scores than younger ones in all sub-scales and items. The older crew members also had higher Standard Deviations as their proficiency is not as consistent as with the younger population. A ceiling effect could be identified, as also identified by Moret-Tatay et al.^23^ The ceiling effect refers to a situation where a measurement or assessment reaches a point where it can no longer increase, even if the underlying ability or attribute continues to improve.^25^

Europeans scored significantly higher in all sub-scales and items than non-Europeans, with the largest difference in the sub-scale “*Calendar*.” This may be attributed to the distribution of officers and non-officers among the cultural groups, with Europeans being strongly associated with the rank as an officer, which will be addressed later. However, cultural differences in subscriber penetration (amount of people who have subscribed to a mobile service) could also be identified by the GSMA SMA Intelligence report “The Mobile Economy 2021.”^26^ According to this report, the subscriber penetration in Europe is 86%, while in Asia Pacific (*including Australia, Southeast Asia, and other Asian countries*) the subscriber penetration is only 56%. Even though mobile service subscription is no direct indicator for mobile proficiency a correlation might be assumed and might therefore pose an explanation for the significant differences in the MDPQ-16 score found in this study.

Similarly, significant differences were found in almost all sub-scales and items between downloaders and non-downloaders, with downloaders performing significantly better—as expected. This may be because people who already have higher mobile proficiency also use health apps more frequently, or because frequent use of these apps leads to a training effect in the use of mobile devices.

With regards to the occupational data, the present findings suggest that the MDPQ-16 sum-score followed the gradient of the degree of education, with the officers (especially the nautical officers) and cadets having higher sum-scores than the ratings with the deck ratings having the lowest average sum-score.

Compared to ratings, officers scored significantly higher on almost every sub-scale and item. As already mentioned, this might partially be due to cultural differences. Being European was strongly associated with the rank as an officer. On the other hand, there might also be socio-economic/educational factors involved. It could be observed that sub-scales with more managerial tasks (eg, *Calendar, Communication, Data/file storage, Software management*) showed higher differences. This might be due to an occupational training benefit of the officers as managerial tasks (*Calendar, Communication e.g.*) have a strong work-related connection. Additionally, nautical officers in training have many digitally based subjects such as computer science, technical navigation, or telecommunications, which could contribute even more to the observed greater competence in mobile proficiency.^27^ This would also explain why cadets scored almost as high as the officers, despite the fact that they could not yet benefit as much as the officers from the occupational training of those tasks.

Additionally, the sub-scale “*Communication*” only ascertained the communication skill via *email.* This probably explains why ratings scored lower than officers as they tend to use messenger apps more frequently to contact their family members according to the experience of the authors of the present study. Emails, on the other hand, are common means of communication in official message exchange from ship to shore, with officers being particularly involved and trained in this regard as part of their reporting obligations.

As already mentioned, sub-scales for simple tasks (*Basics e.g.*) showed rather low score differences between officers and ratings. Additionally, items for private use (eg, *Entertainment*) showed little to no significant difference (“*I can listen to music*”). This might be due to the fact, that those items and skills are of high importance for a remote working collective. As there seems to exist a high demand for listening to music and entertainment, the seafarers developed a higher proficiency in those areas of mobile device usage.

Those tasks also pose an excellent pivot point as especially those skills seem to be a major prerequisite for app-based health intervention. “*Basics*” are needed for operating and navigating health apps, and “*Entertainment*” is needed for orientation/searching for entertainment offers. Another important sub-scale that might play a key role in health app usage is the sub-scale “Internet” (especially the item “I can find health information on the Internet”) which is necessary to search for health-related content for example, health apps. In this sub-scale a bigger difference between officers and ratings of 0.34 points was identified than in the sub-scales of “*Entertainment*” and “*Basics*” (Δ 0.14-0.22). To narrow this gap, IT training could be offered on board by shipping companies, particularly for seafarers with low IT competence. Important topics such as searching, selecting, and evaluating health apps and health information online could be taught. General training in using mobile devices with regard to the benefits of health apps could be particularly helpful for older or less skilled crew members.

### Comparison to the Reference Sample

The seafarers below 65 years scored significantly higher on average by 0.21 points than the Spanish reference population by Moret-Tatay et al.^23^ This is surprising given the fact that due to a prolonged time being offline the use of mobile devices might be limited, which might have raised the assumption of lower frequency and duration of mobile device use and therefore lower proficiency. However these difficulties might be offset by limited alternative communication options of those working at sea. These circumstances most likely do have a promoting effect on the engagement with mobile devices as a sole means of communication with those at home. Moret-Tatay et al,^23^ pointed out that their Spanish sample scored lower than samples surveyed in previous literature. The younger adults (<65 years) in the American study by Roque and Boot^22^ for example scored 4.8 in the MDPQ-16. Thus, the results of the seafarers being more mobile proficient than the sample of Moret-Tatay et al^23^ might not be interpreted too enthusiastically. However, it has to be borne in mind that the younger adults in the study by Roque and Boot^22^ only consisted of 40 individuals and might therefore limit the validity of the results.

Nevertheless, the findings of this survey indicate that the target group of seafarers are sufficiently media-literate for an app-based health intervention, as all displayed tasks had an average mean of between 4 and 5 among the respondents, suggesting that the seafarer sample is on average able to perform the given tasks “reasonably easily” (4) to “very easily” (5). The observation of seafarers performing better than the land-based sample can be explained due to the fact that seafarers have fewer social contacts on board and are separated from their families for months, which means that IT technology might have a higher priority despite the fact of limited online usage^28^ resulting in a higher proficiency due to frequent usage.

A thorough examination of the existing literature unveiled that the MDPQ-16 is among the few validated questionnaires for appraising IT proficiency. Nonetheless, whenever using a questionnaire as the scientific tool for examination, the question remains whether a disparity exists between this “self-reported” proficiency and verified IT proficiency. In the future analytical observation studies that focus on the difficulties of seafarers using health apps and the effects of an intervention on the welfare of seafarers, could provide a precious added value and an even more accurate assessment of mobile proficiency as well.

## Limitations

The questionnaire was limited to self-report measures and only included seafarers on board of one German shipping company. Consequently, it is unclear if the present study findings can be transferred to other seafaring populations. The results are only representative for male sailors, as the data from the 6 women, compared to more than 900 men, cannot have a statistical influence that is reflected in the results.

Cultural, contextual and gender differences (high ratio of men in the seafaring population) have the potential to be a limiting factor. Even though the comparative study used for reference was only 2 years old at the time of data collection, the rapidly evolving nature of digital content and its platforms can introduce changes that might further constrain comparability. This factor should be taken into consideration when interpreting and generalizing the results of this study in relation to the reference data. Additionally, cross-sectional data does not allow for cause-effect interpretations.

## Conclusions and Further Outlook

As shown in this study the seafarers posed a sufficiently mobile/IT proficient sample, even outperforming a Spanish land-based sample, indicating that the seafarers’ technical competence is sufficient enough for health app-based health promotion.

While this study identifies overcoming the proficiency hurdle, it does not explicitly point out that implementing such measures on the high seas could face a variety of other obstacles. This indicates that upcoming research efforts should not solely aim to enhance technological competence, but also to comprehend and address the various other impediments, ranging from limited leisure time to economic and linguistic hurdles or missing awareness of the importance of health support in seafarers.^29^ The accessibility of internet connections on ships remains another significant factor that influences the implementation and usage of health apps. However, the limitation of offline availability may not be as significant a hurdle as it once was, as numerous shipping companies are now in the process of providing internet services on their merchant ships, making an app-based health promotion an even more promising approach. Widespread availability of internet connections is expected to become standard on modern ships, including in seafarers’ cabins. Despite the high IT competence of the collective, training should be offered by shipping companies to accommodate those with weaker IT skills for example, older crew members. Additionally, shipping companies could commission health professionals to determine the specific needs of a remote working collective for app-based health promotion, such as health app topics. In addition, they could identify the needs and knowledge of different cultural groups and ranks. Furthermore, shipping companies should provide their crews with lists of high-quality apps as a recommendation to further enhance the motivation of health app usage. An incentive system could additionally motivate seafarers to use health apps. A solution for financing the usage within the framework of a promotion program offered by the shipping company might help economically less well positioned seafarers with the usage of paid apps.

Overall, an app-based health intervention could be a promising additional long-term measure to promote health, offering a broad range of health topics and different approaches to improve the health of seafarers on board but also on land, as the unhealthy lifestyle does not exclusively occur on board.^15^ In this regard, health promotion apps that can be used beyond the maritime environment would be a great opportunity to mitigate and alleviate the changes in health behavior between these 2 lifestyles. This would also be in the interest of shipping companies, as they would benefit from having healthy and balanced employees.

Expanding on our theoretical basis, we firmly believe that the disparities in IT proficiency among different ranks, as identified in our study, will form a crucial foundation for future research endeavors. This, in turn, will allow for the consideration of rank-related variations in IT competence when implementing electronic health promotion on board. This underlines the need to consider differences in IT competence when implementing eHealth promotion on board.
